# Patient-Reported Outcomes of Dental Implants in Type 2 Diabetes: A Cross-Sectional Study on Quality of Life and Satisfaction

**DOI:** 10.7759/cureus.78091

**Published:** 2025-01-27

**Authors:** Umashree Davangere, Eram Khan, Hiba Chaudhary, Shahinwaz Mulani, Sharanamma B, Seema Gupta

**Affiliations:** 1 Department of Prosthodontics, Pandit Deendayal Upadhyay Dental College, Solapur, IND; 2 Department of Prosthodontics, Sharda School of Dental Sciences, Greater Noida, IND; 3 Department of Public Health Dentistry, ITS Dental College, Ghaziabad, IND; 4 Department of Prosthodontics, Guru Gobind Singh Dental College and Research Center, Burhanpur, IND; 5 Department of Periodontics, Mithila Minority Dental College and Hospital, Darbhanga, IND; 6 Department of Orthodontics, Kothiwal Dental College and Research Centre, Moradabad, IND

**Keywords:** dental implants, diabetes mellitus type 2, patient satisfaction, quality of life, questionnaire

## Abstract

Introduction: Dental implants provide a durable solution for missing teeth and improve mastication, speech, and quality of life (QoL). However, systemic conditions, such as type 2 diabetes mellitus (T2DM), may affect implant success. This study evaluated patient satisfaction (PS) and QoL as patient-reported outcome measures (PROMs) following dental implant therapy in T2DM patients.

Materials and methods: A cross-sectional study was conducted in the Department of Prosthodontics on 90 T2DM patients who had undergone single-unit dental implant therapy at least one year prior to the study. Data were collected using validated PROMs tools, including the Oral Health Impact Profile-14 (OHIP-14) questionnaire for QoL and a nine-item PS questionnaire. Statistical analyses, including regression and mediation, were performed to identify predictors and relationships among the variables.

Results: The study reported moderate QoL scores (mean: 2.71 ± 0.61) and high PS scores (mean: 2.96 ± 0.44). Significant predictors of QoL included sex, implant duration, and implant survival, with successful implants showing a substantial positive association. Implant survival had the strongest direct impact on satisfaction, with ceramic crowns and anterior tooth positions being associated with higher scores. Mediation analysis revealed that factors such as crown type, duration of diabetes, and tooth loss period indirectly influenced outcomes. Implant survival remains the key determinant of both QoL and PS.

Conclusion: T2DM patients undergoing dental implant therapy exhibit moderate QoL and high PS, with implant survival being the most critical predictor of outcomes. Additional factors such as prosthetic choice, tooth position, and systemic health also play significant roles. These findings underscore the importance of glycemic control, individualized care, and robust implant planning to optimize outcomes in T2DM patients.

## Introduction

Dental implants offer a durable and aesthetically pleasing solution for individuals with missing teeth. Beyond functional benefits, such as improved mastication and speech, dental implants are increasingly recognized for their potential to enhance patients' quality of life (QoL) and patient satisfaction (PS) [1]. Nevertheless, the efficacy and results of dental implant procedures can be affected by systemic health issues, with type 2 diabetes mellitus (T2DM) being of particular significance. T2DM is a persistent metabolic condition marked by elevated blood glucose levels, which is linked to numerous complications that may affect oral health, such as deterioration of periodontal health, delayed wound healing, increased chances of bone resorption, and failure of osseointegration, jeopardizing the success of dental implant procedures [2].

The estimates in 2019 showed that 77 million individuals had diabetes in India, which is expected to rise to over 134 million by 2045 [3]. This demographic transformation emphasizes the importance of understanding the interaction between T2DM and dental implant outcomes. Notwithstanding these obstacles, progress in implant materials, surface enhancements, and surgical methodologies has rendered implant therapy a feasible alternative for T2DM individuals under appropriate glycemic management. Consequently, it is essential to evaluate the broader ramifications of dental implant therapy in T2DM individuals, particularly concerning their PS and QoL [4,5].

PS and QoL are complex constructs shaped by an array of physical, psychological, and social determinants. In the context of individuals with T2DM, oral rehabilitation via dental implants transcends mere functional restoration; it can mitigate the psychological distress associated with tooth loss and bolster social self-esteem. Evaluating these outcomes yields essential insights into the comprehensive advantages of dental implant therapy and facilitates the customization of treatment strategies to address the specific requirements of T2DM patients [6,7]. Moreover, patient-reported outcome measures (PROMs) have become increasingly significant in clinical research, serving as essential instruments for assessing treatment effectiveness from the patient’s perspective. Such measures are especially pertinent in chronic illnesses, such as diabetes, where individual experiences and health-related QoL substantially influence overall wellness [8].

Comprehending PROMs such as PS and QoL is pivotal for enhancing the efficacy of care provision and guaranteeing that therapeutic approaches are congruent with patient anticipation and inclination. Furthermore, examining the potential disparities in outcomes between diabetic and non-diabetic cohorts can yield significant insights into the influence of systemic conditions on the perceived advantages of dental implant interventions [6]. This study aimed to evaluate PROMs, such as PS and QoL outcomes, following dental implant procedures in patients diagnosed with T2DM. By employing validated PROMs and analyzing the factors influencing these results, this study seeks to rectify existing knowledge gaps and contribute to the growing body of evidence that supports patient-centered methodologies within restorative dental care.

## Materials and methods

Study design and setting

This cross-sectional study was conducted in the Department of Prosthodontics, Sharda School of Dental Sciences, Greater Noida, India, from April 2023 to December 2023. The requirement for ethical committee approval was waived by the Institutional Ethical Committee of Sharda School of Dental Sciences, as no identifying or sensitive information was used in the study. The data were anonymized, and confidentiality was maintained. No identifying information has been included in the manuscript for publication. The study adhered to the principles of the Declaration of Helsinki, and written informed consent was obtained from all participants before starting the study. Participants were informed of their right to withdraw from the study at any time, without repercussions. No financial incentives were offered to ensure voluntary participation in the study.

Study population

The study included diabetic patients with T2DM who had undergone single-unit dental implant therapy with a follow-up period of at least one year prior to recruitment. The eligibility criteria included adults aged 18 years and older, a confirmed diagnosis of T2DM, in a state of satisfactory overall health as per the American Society of Anesthesiologists (ASA) classification, specifically ASA I or II, requiring implant-prosthetic rehabilitation within the anterior and/or posterior regions of the maxillary or mandibular jaw to replace a missing tooth with an extraction history of at least six months, and possessing sufficient oral hygiene as indicated by a Simplified Oral Hygiene Index (OHI-S) score of less than 3 [9]. Patients with uncontrolled diabetes (glycated hemoglobin, HbA1c > 8%), ongoing periodontal infections, pregnant and lactating females, systemic conditions contraindicating implant placement, smokers, tobacco chewers, alcoholics, or cognitive impairments that could hinder survey participation were excluded. All patients underwent two-stage implant placement (Adin Dental Implant Systems Ltd., Afula 1811101, Israel) following a standardized protocol by the same clinician with more than five years of experience (UD). Two other clinicians performed the data collection (EK, SM).

Sample size estimation, sampling, and recruitment

The participants were recruited from the patient database of the Department of Prosthodontics. Invitations were sent via phone and email to report for follow-up. G Power software (Heinrich-Heine-Universität Düsseldorf, Düsseldorf, Germany) was used for sample size estimation. A minimum of 90 patients was estimated considering an effect size of 0.52 with a mean difference of 0.03 in overall QoL scores in diabetic and non-diabetic patients rehabilitated with dental implants, and a pooled SD of 0.05 [10]. The power of the study was 80% with an alpha error of 5%.

Data collection

Records from January 2017 to March 2022 were searched. During this period, a total of 835 patients received dental implants in the Department of Prosthodontics; however, only 90 patients who fulfilled the eligibility criteria were called for follow-up. Data were collected using a combination of clinical examinations and self-administered questionnaires. Demographic and clinical data were obtained from a standardized case report form used in the department for implant cases to collect demographic information such as age, sex, education level, and clinical data such as duration of diabetes, reason for missing tooth, duration of extraction, HbA1c levels, location of implants, and time since implant placement. The survey assessed the satisfaction levels of participants using a validated questionnaire developed by Kim et al. [11]. This study employed nine inquiries pertaining to patient contentment, specifically focusing on aspects such as masticatory function, verbal communication capabilities, comfort associated with implants, ease of cleaning, aesthetic appearance, the surgical implantation process, maintenance procedures, associated costs, and overall usability, scored using a five-point Likert scale ranging from completely unsatisfied to completely satisfied. QoL outcomes were evaluated using the Oral Health Impact profile-14 (OHIP-14) questionnaire [12], a widely used validated instrument that measures the impact of oral health on functional limitation, physical pain, psychological discomfort, physical disability, psychological disability, social disability, and handicap. Each domain comprised a pair of inquiries evaluated on a five-point grading scale: 0 = never; 1 = almost never; 2 = occasionally; 3 = often; and 4 = very often or daily. Domain scores were calculated by aggregating responses to the two inquiries. The total scores were derived by summing all scores from the 14 inquiries. The scoring spectrum ranged from 0 to 56, whereas the domain scores varied from 0 to 8. An elevated OHIP-14 score indicates a more detrimental impact on oral health-related QoL, and glycemic control was assessed using recent HbA1c levels obtained from patients' medical records. HbA1c levels were categorized as controlled (≤7%) and uncontrolled (>8%). The clinical examination was conducted by an experienced periodontist (SB) to assess implant health, including probing depth and bleeding on probing.

Statistical analysis

The collected measurements were systematically analyzed using SPSS software (IBM Corp. Released 2013. IBM SPSS Statistics for Windows, Version 23.0. Armonk, NY: IBM Corp.) by a statistician (SG) who was blinded to the allocation of groups. Descriptive statistics, including frequency distributions, means, and SD were used to summarize the data. Regression analysis was performed to predict the independent variables affecting the outcome measures. Furthermore, PS and QoL scores were analyzed using a mediation analysis. Statistical significance was set at a threshold of P<0.05.

Reliability and validity

The reliability of the OHIP-14 and PS questionnaire was assessed using Cronbach’s alpha, with a threshold of ≥0.8 considered acceptable. Validity was ensured through a pilot study involving 10 T2DM patients who provided feedback on questionnaire clarity and relevance. Minor revisions have been made based on their input.

## Results

The most common reasons for tooth loss were periodontal disease and failed root canal treatment in 33 (36.67%) patients; males and females were equally represented to prevent bias due to sex. Dental implants were successful in 72 (80%) patients and failed in 18 (20%) patients. Ceramic crowns were preferred by 54 (60%) patients. Bone grafts were used in 25 (27.77%) patients. These findings suggested that endodontic and periodontal issues are the primary contributors to tooth loss, and ceramic crowns and implants had favorable outcomes (Table 1).

**Table 1:** Descriptive analysis of study variables. Data is presented in the form of n (%). RCT: root canal treatment; PFM: porcelain-fused-to-metal

Variables	Category	Frequency	Relative frequency (%)
Reason for tooth loss	Periodontal	33	36.67%
	Failed RCT	33	36.67%
	Periapical cyst	15	16.67%
	Trauma	9	10.00%
Sex	Male	45	50.00%
	Female	45	50.00%
Implant status	Success	72	80.00%
	Failed	18	20.00%
Crown material	Ceramic	54	60.00%
	PFM	36	40.00%
Bone graft	Yes	25	27.77%
	No	65	72.23%

The mean age of the participants was 42.97 ± 6.96 years. The duration of diabetes averaged 54.00 ± 15.50 months, while the mean duration of the implant use was 2.83 ± 0.90 years. The average period of tooth loss was 13.2 ± 3.01 months. The mean QoL and PS scores were 2.71 ± 0.61 and 2.96 ± 0.44, respectively. These results indicated moderate QoL and high PS, with diabetes duration and age being important variables for implant outcomes (Table 2).

**Table 2:** Inferential statistics for the study parameters. Data is presented in form of mean ± SD. QoL: quality of life; PS: patient satisfaction; CI: confidence interval

Variables	Minimum	Maximum	95% CI for mean	Mean ± SD
Age (years)	32	56	41.51 - 44.42	42.97 ± 6.96
Duration of diabetes (months)	24	96	50.75 - 57.25	54.00 ± 15.50
Duration of implant (years)	2	5	2.64 - 3.02	2.83 ± 0.90
Period of tooth loss (months)	8	18	12.57 - 13.83	13.2 ± 3.01
QoL score	1.44	3.28	2.58 - 2.84	2.71 ± 0.61
PS score	1.89	3.44	2.87 - 3.05	2.96 ± 0.44

Significant predictors of QoL included sex, implant duration, and implant survival. Male participants showed significantly lower QoL than did female participants. A longer implant duration positively impacted QoL. Implant survival was the strongest predictor, with successful implants showing a substantial association with an improved QoL. These results emphasized that implant success is the key determinant of improved QoL (Table 3).

**Table 3:** Regression analysis for QoL. *P-value <0.05 is considered significant. PFM, porcelain-fused-to-metal; QoL, quality of life

Model	Coefficients beta	Standard error	t-value	P-value	95% confidence interval for B	
					Lower bound	Upper bound
Age	0.07	0	1.79	0.078	0	0.01
Sex (male vs. female)	-0.17	0.04	-5.77	0.001*	-0.28	-0.14
Duration of diabetes in months	-0.05	0	-1.17	0.147	0	0
Duration of implant in years	0.02	0.02	0.96	0.042*	-0.05	-0.02
Bone graft (yes vs. no)	0.03	0.01	0.34	0.128	-0.01	0.03
Implant survival (success vs. failure)	1.02	0.04	41.15	0.001*	1.47	1.62
Period of tooth loss in months	0.02	0	1.06	0.219	0	0.01
Type of crown (ceramic vs. PFM)	0.03	0.05	0.93	0.215	-0.05	0.13
Tooth position (posterior vs. anterior)	-0.01	0.03	-0.25	0.319	-0.08	0.06
Tooth type (molar vs. incisor)	0.01	0.06	0.23	0.818	-0.11	0.13

**Figure 1:**
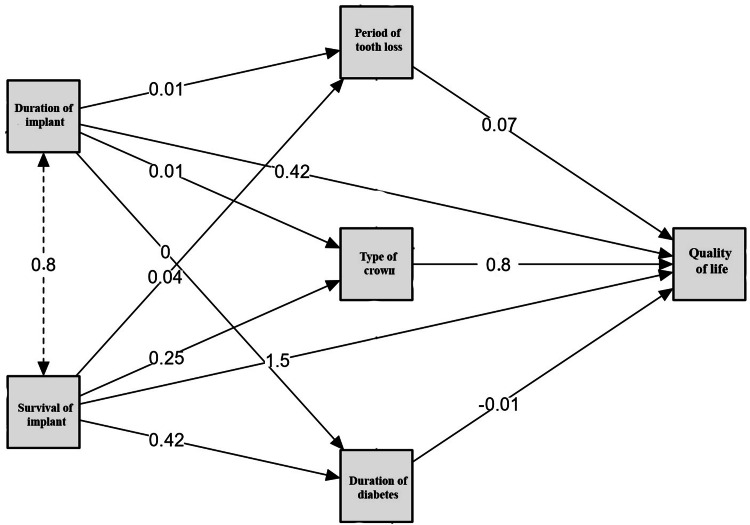
Mediation analysis for QoL. Predictive factors (survival of implant and duration of implant); mediators (duration of diabetes, duration of tooth loss, and type of crown); outcome (QoL score). QoL, quality of life

Mediation analysis demonstrated the influence of implant survival and implant duration on QoL. Implant survival significantly impacted QoL directly (0.8) and indirectly through the type of crown (0.8), period of tooth loss (0.07), and duration of diabetes (-0.01). The duration of the implant also contributed indirectly via the type of crown (0.42) and period of tooth loss (0.01). Implant survival exerted a stronger overall effect than the duration of the implant, emphasizing its critical role. These findings highlight that while implant survival is key, intermediate factors such as prosthetic choice and systemic health mediate its overall influence on the QoL of the patients (Figure 1).

Significant predictors of PS included age, sex, implant survival, crown type, and tooth position. Older age was associated with reduced satisfaction, whereas males reported higher satisfaction than females. Implant survival had the strongest positive effect on PS. Ceramic crowns are more favorable than porcelain-fused-to-metal (PFM) crowns. Tooth position also impacted satisfaction, with incisors being rated higher. Factors such as duration of diabetes and bone grafting showed no significant effects (Table 4).

**Table 4:** Regression analysis for PS. *P-value <0.05 is considered significant. PFM, porcelain-fused-to-metal; PS, patient satisfaction

Model	Coefficients beta	Standard error	t-value	P-value	95% confidence interval for B	
					Lower bound	Upper bound
Age	-0.41	0.01	-5.08	0.001*	-0.04	-0.02
Sex (male vs. female)	0.19	0.05	3.1	0.003*	0.06	0.27
Duration of diabetes in months	0.12	0	1.51	0.136	0	0.06
Duration of implant in years	-0.06	0.02	-1.21	0.229	-0.08	0.02
Bone graft (yes vs no)	-0.13	0.06	1.34	0.233	-0.12	0.18
Implant survival (success vs. failure)	0.87	0.05	17.5	0.001*	0.85	1.06
Period of tooth loss in months	-0.14	0.01	-3.08	0.103	-0.03	0.01
Type of crown (ceramic vs. PFM)	0.1	0.07	1.4	0.016*	0.04	0.22
Tooth position (posterior vs. anterior)	-0.03	0.05	-0.58	0.013*	-0.13	-0.07
Tooth position (molar vs. incisor)	-0.17	0.09	-2.08	0.041*	0.01	0.35

**Figure 2:**
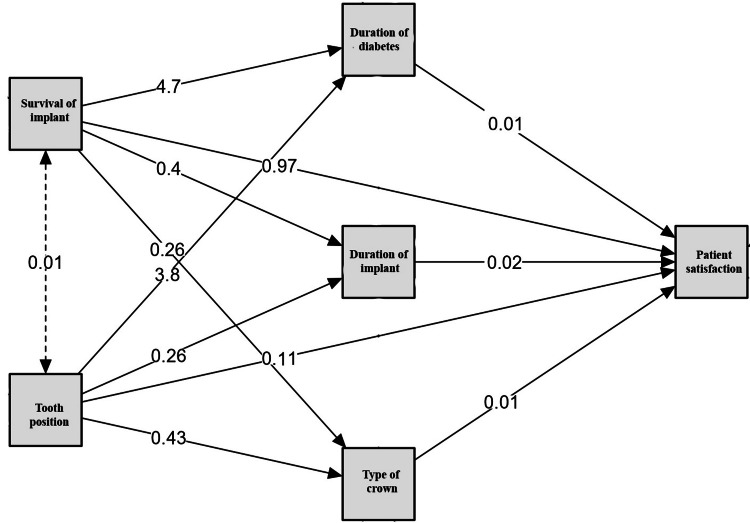
Mediation analysis for PS. Predictive factors (survival of implant and tooth position); mediators (duration of diabetes, duration of implant, and type of crown); outcome (PS score). PS, patient satisfaction

Mediation analysis highlighted the complex relationships between implant survival, tooth position, and PS. Implant survival directly and positively influenced PS, with pathways mediated through variables such as crown type (0.01), implant duration (-0.02), and tooth position (0.26). Tooth position also affected PS indirectly through the type of crown (0.11) and duration of implant. Implant survival had a stronger direct effect (4.7) on PS than tooth position (p = 0.01). These results emphasize that while implant survival is critical, factors such as tooth position and prosthetic type contribute indirectly, underscoring the multifactorial determinants of PS (Figure 2).

## Discussion

This study evaluated the outcomes of dental implant therapy in individuals with T2DM, focusing on PS and QoL as PROMs. The findings provide valuable insights into the interplay between systemic health conditions such as T2DM and the clinical and psychological benefits of dental implant therapy. The findings underscore notable correlations between implant longevity and PROMs, highlighting implant durability as the primary predictor of both QoL and PS, as corroborated by Duong et al. [13]. Implant sustainability was directly associated with enhanced QoL and increased PS, with mediating indirect effects arising from variables such as crown design, dental position, and systemic health considerations, including the duration of diabetes. These results align with the current body of literature that emphasizes the essential importance of effective osseointegration and durability of implants in realizing positive clinical and psychological outcomes in dental implant treatment [6,14].

Quality of life outcomes

The investigation demonstrated moderate QoL assessments among the subjects, with notable determinants including sex, longevity of the implant, and duration of its presence. Female participants exhibited superior QoL results compared to their male counterparts, a result that may be ascribed to variances in psychological and social coping strategies and anticipations [15]. Additionally, the long-term stability of implants enhances oral functionality and mitigates the psychological distress associated with tooth loss [16]. Herrero et al. [17] concluded that dental implant therapy has demonstrated considerable enhancements in patient-reported advantages associated with mandibular two-implant overdentures among individuals with T2DM, comparable to the benefits observed in healthy edentulous populations. Notably, these advantages are also applicable to individuals who exhibit poorly regulated glycemia.

Interestingly, the mediation analysis demonstrated that systemic health factors, such as the duration of diabetes and the period of tooth loss, exerted indirect effects on QoL. The negative impact of prolonged diabetes duration on implant success and subsequent QoL underscores the importance of glycemic control for optimizing treatment outcomes. However, findings from a single-center, prospective cohort investigation conducted by Oates et al. [18] indicated that increased HbA1c concentrations in individuals diagnosed with T2DM did not correlate with modified implant survival rates one year after loading.

Patient satisfaction outcomes

The elevated PS scores identified in this study underscored the efficacy of dental implant interventions in fulfilling patient anticipations. Notable determinants of satisfaction encompassed variables such as age, sex, implant longevity, crown type, and dental location. Older age was associated with reduced satisfaction, possibly due to age-related changes in oral and systemic health that may influence treatment perceptions [19]. The inclination toward ceramic crowns as opposed to PFM crowns further emphasizes the critical role of prosthetic materials in shaping patients' perceptions of treatment efficacy [20]. Ceramic crowns are frequently linked with enhanced aesthetic qualities and longevity, which likely accounts for the elevated satisfaction ratings noted in this investigation [20]. Additionally, the position of the teeth exerted a notable impact on satisfaction levels, with anterior implants receiving higher ratings attributable to their visible aesthetic influence and significance in social engagement [21]. The longevity of implants exhibits the most significant direct influence on PS, aligning with its function in guaranteeing both functional and aesthetic efficacy [17]. Nevertheless, mediation analysis indicated that intermediary factors, including crown type and implant duration, were also pivotal contributors. These results underscore the complex and multifaceted characteristics of PS, which transcends mere clinical outcomes including psychological and social aspects.

Clinical implications

The robust correlation between implant longevity and PROMs highlights the criticality of securing and preserving implant stability. This demands a comprehensive treatment strategy that encompasses judicious patient selection, meticulous surgical execution, and suitable prosthetic decisions. The incorporation of sophisticated implant materials and surface enhancements may additionally improve osseointegration and long-term viability, especially in individuals with systemic disorders such as T2DM. The research underscores the necessity for individualized therapeutic approaches that consider distinct patient attributes, such as age, sex, and overall health condition. For instance, geriatric patients might gain from enhanced assistance and guidance to tackle age-specific issues, whereas female patients may require interventions customized to their aesthetic and psychological requirements, which underscores the critical role of glycemic regulation in enhancing implant results for individuals with T2DM. Diligent surveillance of HbA1c levels and proficient management of systemic health issues should constitute the fundamental elements of both preoperative and postoperative care. Educating patients regarding the influence of glycemic regulation on implant success and QoL can further optimize treatment compliance and outcomes.

Limitations of the study

The cross-sectional methodology inhibits the capacity to ascertain causal relationships among the variables. Longitudinal research is required to assess the enduring effects of implant therapy on QoL and satisfaction in patients with T2DM. Furthermore, dependence on PROMs may introduce response bias, although the implementation of validated instruments alleviates this issue to a certain degree. The study's concentration on a single center may restrict the applicability of its findings to diverse settings and populations. Future investigations should encompass multicenter trials with larger sample sizes to corroborate the results and examine possible regional and cultural variances in treatment outcomes. Finally, while the study highlights the significance of implant longevity and prosthetic selection, additional aspects, such as patient education, psychological assistance, and postoperative management, were not explicitly analyzed. Future studies should explore these factors to create holistic care models that cater to the diverse requirements of T2DM patients.

## Conclusions

This study reported moderate QoL and high PS scores in patients with T2DM. This underscores the critical role of implant survival in determining QoL and PS in patients with T2DM undergoing dental implant therapy. The findings highlight the multifactorial nature of treatment outcomes, with significant contributions from systemic health factors, prosthetic choices, and patient demographics. Significant predictors of QoL included sex, implant duration, and implant survival, and significant predictors of PS included age, sex, implant survival, crown type, and tooth position.
